# Integrating Molecular Docking, Dynamics, and AI-Based ADMET Prediction to Decipher the Toxicity Profile of Isometamidium Chloride Against Animal Trypanosomiasis

**DOI:** 10.3390/jox16060179

**Published:** 2026-09-22

**Authors:** Shalini Batheja, Shalki Choudhary, Swati Rani, Rajender Kumar, Balvinder Kumar, Anju Manuja

**Affiliations:** ICAR-National Research Centre on Equines, Hisar 125001, India

**Keywords:** isometamidium chloride, machine learning tools, toxicity

## Abstract

Isometamidium chloride (ISM) is a widely used trypanocidal drug for treating animal trypanosomiasis, despite concerns about toxicity, and emerging drug resistance. A comprehensive in silico evaluation integrating molecular interaction analysis, pharmacokinetic profiling, toxicity prediction, and molecular dynamics simulations has not yet been reported. Owing to the unavailability of a crystal structure for *Trypanosoma evansi*, the co-crystal structure of the catalytic domain of thymidine kinase from *T. brucei* (5FUW, 2.20 Å) was used for computational studies. ISM exhibited strong binding affinity and stable protein–ligand interactions throughout a 100 ns MD simulation. ADMET analysis predicted high gastrointestinal absorption, potential CYP-mediated interactions, and toxicity risks. Compared with QS, ISM showed better drug likeness, complete compliance with Lipinski’s rule, and a higher predicted bioavailability score. Collectively, these findings demonstrate that although ISM possesses favorable target affinity, its pharmacokinetic and safety limitations warrant structural optimization and safer formulation strategies for improved trypanocidal therapy.

## 1. Introduction

Animal trypanosomiasis is a major parasitic disease that affects livestock productivity across sub-Saharan Africa, Asia, and parts of Latin America, causing significant economic losses from anemia, infertility, reduced milk production, weight loss, and death [1,2]. The disease is caused by several species in the genus Trypanosoma, including *T. congolense*, *T. vivax*, various subspecies of *T. brucei*, and *T. evansi*. However, controlling the insect vectors that transmit these parasites is often difficult due to practical constraints. As a result, drug treatment (chemotherapy) remains the most feasible and widely adopted approach for managing the disease in endemic regions. Therefore, the ongoing effectiveness and safety of available trypanocidal drugs are critically important.

The chemotherapeutic management of animal trypanosomiasis relies on a very limited number of legacy drugs introduced decades ago. Among these, isometamidium chloride (ISM) and quinapyramine sulfate (QS) remain widely used in veterinary practice for their cost-effectiveness and broad-spectrum trypanocidal activity [3]. However, prolonged use has led to increasing reports of treatment failure and reduced drug responsiveness. Field-based studies have documented resistance to commonly used trypanocides, underscoring resistance as a major challenge for sustainable disease control programs [4].

ISM is a cationic trypanocide used for the treatment of the infection. Experimental studies have shown that ISM preferentially accumulates in the kinetoplast DNA (kDNA) of trypanosomes, leading to mitochondrial dysfunction and parasite death [5,6]. However, the same physicochemical properties that make it effective against parasites are also associated with host cell toxicity, which limits its therapeutic window. Strong experimental evidence supports DNA interaction-mediated toxicity of ISM [7].

QS, a quinoline–pyrimidine trypanocide used against *T. evansi*, is often administered alone or in prosalt formulations. Compared with ISM, quinapyramine has distinct physicochemical properties, including greater molecular flexibility and a different charge distribution, which can influence its pharmacokinetic behavior and interactions with biological targets. However, its clinical use is limited by dose-dependent side effects, including toxicity at higher doses, underscoring the need for improved safety profiling [8,9,10].

In recent years, in silico methods have evolved beyond traditional molecular docking to include machine learning (ML)-based predictive models, as well as MD simulations for analyzing interactions over time. These advanced computational tools enable the integration of physicochemical properties and absorption, distribution, metabolism, excretion, and toxicity (ADMET) endpoints [11,12]. The computational evaluation of QS in the recently published study, focusing on toxicity, mutagenicity risk, multi-parameter ADMET, and pharmacokinetics, offers a modern in silico safety profile of QS [10]. To our knowledge, this is the first study to evaluate the therapeutic potential and toxicity profile of ISM. The study aims to use modern in silico tools (1) to assess ADMET features, toxicity risks, and safety of ISMs, providing a blueprint for rational optimization. Further, we address the research questions: (2) How do ISM and QS differ in their predicted binding affinity and interaction profiles with target proteins? (3) What are the key differences in their physicochemical and pharmacokinetic properties that could affect their safety and efficacy? (4) What are the potential toxicity risks predicted using ML-based tools?

## 2. Materials and Methods

### 2.1. Molecular Property and Physicochemical Analysis

ISM is a veterinary trypanocidal drug widely used for the treatment and prophylaxis of animal trypanosomiasis, as shown in Figure 1 [7]. It is a planar, aromatic structure with a strong positive charge that enhances electrostatic and π–π interactions with negatively charged biological macromolecules, especially nucleic acids. Key molecular descriptors, including molecular weight (MW), aromaticity, fraction of Csp^3^, hydrogen-bond donors (HBD) and acceptors (HBA), rotatable bonds, molar refractivity (MR), and topological polar surface area (TPSA), were calculated using SwissADME (2024 version; developed by SIB Swiss Institute of Bioinformatics; Lausanne; Switzerland) and related tools. These parameters help assess structural complexity, polarity, and potential bioavailability [13,14].

### 2.2. Docking Experiments

#### 2.2.1. Receptor Preparation

Since the PDB of *T.evansi* was not available, the co-crystallized structure of *T. brucei* (PDB ID: 5FUW, co-crystallized ligand: QBT, XYZ coordinates: −16.7547, −23.2847, −2.02167) was used. The *T. brucei* protein (5FUW) was selected due to its 89% protein sequence homology with *T. evansi*’s catalytic domain, as explained in our previous study, ensuring biological relevance [10]. Missing residues were modeled, hydrogen atoms were added, water molecules were removed, and Gasteiger charges were assigned [15]. The catalytic pocket was defined using the coordinates of the co-crystallized ligand.

#### 2.2.2. Ligand Preparation

The 2D structure of ISM was converted to a 3D conformation and energy-minimized using the MMFF94 force field [16]. The optimized structure was then converted to PDBQT format for docking. Hydroxychloroquine (HCQ) was used as the reference drug due to its well-established molecular structure, relevant aromatic and ionizable features supporting molecular interactions, and previously reported anti-trypanosomal activity [17]. Therefore, HCQ was considered a suitable comparative reference for the present computational analysis. The docking performance of ISM was evaluated in comparison with QS and HCQ (reference drug).

#### 2.2.3. Docking Procedure

Docking simulations were performed using PyRx (version 0.8; Sargis Dallakyan, The Scripps Research Institute, La Jolla, CA, USA) and visualized with Discovery Studio Visualizer (BIOVIA Discovery Studio Visualizer 2024; Dassault Systèmes BIOVIA; San Diego, CA, USA) [18]. The grid box was centered on the co-crystallized QBT ligand within the catalytic pocket, with a 4 Å margin. The best binding poses were selected based on the lowest binding energy and interaction profile.

### 2.3. Molecular Dynamics Simulation

The best docked pose of ISM was selected for MD simulation using GROMACS 2021.1 (GROMACS Development Team; Stockholm, Sweden) (https://www.gromacs.org/) to assess the stability of the protein–ligand complex under physiological conditions [19,20]. Input files were generated with the Solution Builder module of the CHARMM-GUI (University of Kansas; Lawrence, KS, USA) (https://www.charmm-gui.org/) web server [21]. The protein–ligand complex was solvated in a TIP3P water box, and sodium and chloride ions were added to neutralize the system.

Energy minimization was performed using the steepest descent algorithm for 5000 steps to remove unfavorable steric contacts. Subsequently, the system was equilibrated under NVT (no. of particles, volume and temperature) and NPT (no. of particles, volume and pressure) conditions at 310.15 K with the CHARMM36m force field. Following equilibration, a 100 ns production MD simulation was conducted to investigate the conformational stability and dynamic behavior of the complex.

The generated trajectories were analyzed using standard structural parameters, including root mean square deviation (RMSD), root mean square fluctuation (RMSF), and radius of gyration (Rg), to assess the stability, flexibility, and compactness of the protein–ligand complex. In addition, principal component analysis (PCA) was performed to characterize the dominant collective motions of the protein during the simulation. PCA is a multivariate statistical method based on the covariance matrix of atomic fluctuations and is widely used to identify large-scale conformational motions while reducing the dimensionality of MD trajectory data [22].

Furthermore, the free energy landscape (FEL) was constructed using RMSD and Rg as reaction coordinates to investigate the thermodynamic stability of the complex [23,24]. The FEL provides information on energetically favorable conformational states and the transitions that occur during the simulation. Low-energy basins correspond to highly populated, stable conformations, whereas higher-energy regions represent less favorable structural states.

### 2.4. Lipophilicity and Solubility Prediction

Lipophilicity (Log P) was predicted with multiple models (ILOGP, XLOGP3, WLOGP, MLOGP, and SILICOS-IT) to minimize model-specific bias. Aqueous solubility (Log S) was estimated with the ESOL, Ali, and SILICOS-IT models to assess oral absorption potential, given that poor solubility is a common challenge in veterinary drug development [25,26].

### 2.5. ADMET Data Prediction

Pharmacokinetic parameters, including gastrointestinal absorption, blood–brain barrier permeation, P-glycoprotein interaction, and cytochrome P450 inhibition, were predicted using SwissADME. Toxicity assessment focused on oral toxicity and molecular initiating events, using the ProTox-3.0 platform, which employs AI- based ML models trained on curated toxicological datasets [10,12]. The toxicity classes (Active/Inactive) were reported directly from the ProTox-3.0 output, while the associated probability values were used to indicate prediction confidence. Predictions with probabilities ≥0.7 and <0.7 were interpreted as higher and comparatively lower-confidence predictions, respectively. Although molecular docking was used to evaluate potential interactions, greater emphasis was placed on ML-driven analyses of ADMET and toxicity data. These computational approaches provided insights into pharmacokinetic properties, safety profiles, and potential toxicity risks, thereby supporting a future-oriented framework for trypanocide safety assessment.

## 3. Results and Discussion

### 3.1. Docking Analysis

Docking simulations showed that ISM had a stronger binding affinity of −8.4 kcal/mol than QS, which had −5.6 kcal/mol. HCQ, used as a reference compound as shown in Table 1, displayed the strongest binding affinity with −8.8 kcal/mol [10], as indicated by more negative binding energy values. The greater binding stability of ISM can be attributed to its rigid phenanthridinium core, which promotes extensive π–π stacking interactions and electrostatic complementarity within the active site. In contrast, QS demonstrated a comparatively moderate binding affinity, likely due to its more flexible molecular structure, which may reduce the extent of conformational stabilization within the binding pocket.

#### Interaction Analysis

Interaction profiling revealed distinct binding patterns among the three compounds: ISM (A) forms hydrogen bonds with key residues Asp244, Glu286 and Thr245, participates in π–π stacking with Phe289, and makes hydrophobic contacts with Phe215 and Met214 using Discovery Studio, as illustrated in Figure 2. The rigid, planar structure of ISM allows optimal alignment within aromatic-rich binding regions, enhancing binding stability. Its positively charged centers play a crucial role in electrostatic stabilization, especially in environments with negatively charged active sites. However, ISM shows improved aromatic stacking and electrostatic stabilization compared to QS.

QS (B) exhibited multiple interactions with key amino acid residues, including Met214, Glu286, Phe321, Tyr370, and Leu312. Compared with ISM, QS exhibited a less favorable predicted binding energy (−5.6 kcal/mol), suggesting weaker predicted binding. It demonstrated a balanced but less extensive interaction network than ISM. Its binding stability mainly relies on hydrogen bonding and moderate hydrophobic interactions rather than extensive aromatic stacking. HCQ (C) showed interactions involving Met214, Glu286, Asp244, Phe289, and Leu312, comprising hydrogen-bond formation, moderate hydrophobic interactions and fewer π–π stacking interactions than ISM, as displayed in Table 2. Thus, HCQ exhibited the most favorable predicted binding energy, followed by ISM, whereas QS showed comparatively weaker binding energy.

### 3.2. MD Trajectory Analysis- RMSD, RMSF and Rg

To further validate the docking results and assess the dynamic stability of the protein–ligand complex, a 100 ns molecular dynamics simulation was performed for the ISM–5FUW complex. The RMSD trajectory showed an upward trend during the simulation period, ranging from approximately 0.12 to 0.50 nm with an average of 0.32 nm, indicating that the complex undergoes conformational adjustments before reaching a plateau, as seen in Figure 3. Compared with the previously reported QS complex (0.18–0.42 nm) and HCQ complex (0.20–0.60 nm), the ISM complex exhibited comparable overall fluctuations and reached a stable structural state. Rg varied between 1.61 and 1.70 nm with an average of 1.66 nm, demonstrating preservation of structural compactness and the absence of major unfolding events, despite a slight compaction observed after 650 frames. Residue-wise flexibility analysis revealed low fluctuations across most amino acid residues, with only a few localized peaks around residues 48–55 and a sharp pair of peaks between residues 160–180 reaching up to 0.60 nm, indicating that ligand binding maintained the overall stability of the receptor framework with specific flexible loops. Principal component analysis (PCA) cumulative explained variance showed that the first two components capture over 55% of the total variance, and the motion is mostly accounted for by eight components. The Cartesian coordinate PCA projection showed a structured conformational distribution along the first two principal components, indicating distinct stable conformational states during the simulation period. Similarly, the free energy landscape (FEL) exhibited a well-defined low-energy basin, suggesting the presence of a dominant thermodynamically favorable conformational state during the 100 ns trajectory.

Previous MD studies on QS and HCQ also reported stable conformational behavior; however, the ISM complex exhibited a more compact conformational distribution and a well-defined energy minimum, indicating enhanced dynamic stability. Collectively, RMSD, RMSF, Rg, PCA, and FEL analyses consistently showed that ISM forms a stable, energetically favorable complex with the target protein throughout the 100 ns simulation, supporting the docking results and indicating that ISM’s rigid aromatic scaffold contributes to stronger, more persistent receptor interactions than QS and HCQ.

### 3.3. Physicochemical Property Analysis

The in silico evaluation of physicochemical properties revealed significant differences between ISM and QS. ISM exhibited high aromaticity and a low fraction of sp^3^-hybridized carbon atoms, indicating a rigid, planar molecular structure as presented in Figure 4. These features are known to enhance strong π–π stacking and electrostatic interactions with nucleic acids, while also reducing molecular flexibility and permeability. Table 3 illustrates molecular descriptors and physicochemical properties of ISM and QS. It revealed that higher topological polar surface area (TPSA) values further suggest poor membrane permeability of ISM, consistent with established structure–property relationships for cationic aromatic compounds [13].

In contrast, QS exhibited greater molecular flexibility and lower aromaticity, suggesting distinct interaction patterns with biological macromolecules and potentially improved permeability. Increased molecular flexibility is associated with greater conformational adaptability but may also lead to off-target interactions, which is important for toxicity prediction.

### 3.4. Lipophilicity and Solubility Prediction

Lipophilicity predictions from multiple computational models (ILOGP, XLOGP3, WLOGP, MLOGP, and SILICOS-IT) as shown in Table 4 indicated that both ISM and QS have suboptimal lipophilicity profiles for oral administration. ISM consistently showed poor aqueous solubility across the ESOL and SILICOS-IT models, a known limitation of highly charged aromatic compounds that directly affects gastrointestinal absorption and systemic exposure [25,27].

QS showed slightly better predicted solubility than ISM; however, solubility values remained within a low-to-moderate range. Previous computational and experimental studies have emphasized that poor solubility is a key factor in variable pharmacokinetics and therapeutic inconsistency in veterinary trypanocides [28].

### 3.5. ADMET and Oral Toxicity Profiling

ML-enabled ADMET prediction revealed distinct toxicity risk profiles for ISM and QS. ISM showed high predicted gastrointestinal absorption, no blood–brain barrier permeation, and a tendency to inhibit cytochrome P450 enzymes, indicating potential drug–drug interaction risks as depicted in Table 5. ML-based oral toxicity prediction, as displayed in Table 6, showed ISM in higher toxicity risk categories, reinforcing concerns about its narrow therapeutic margin. These computational toxicity warnings align with previous reports linking aromatic cationic compounds to increased systemic toxicity risk [29].

QS showed a unique ADMET profile with predicted warnings for oral toxicity and mutagenicity. The in silico study Batheja et al. (2025) also reported an oral toxicity risk and pharmacokinetic limitations for QS, despite acceptable interaction scores, highlighting that toxicity still limits systemic use [10]. This comparative analysis supports these results and points out safety issues for both compounds. The radar plot in Figure 5 illustrates the comparative toxicity profiles, highlighting variations across multiple biological targets and safety parameters. Although ISM was predicted to be inactive for hepatotoxicity and cytotoxicity, it showed potential toxicity concerns for other endpoints, including neurotoxicity, respiratory toxicity, carcinogenicity, and mutagenicity.

An apparent discrepancy was observed between the BBB-related predictions obtained from SwissADME and ProTox-3.0. SwissADME predicted both ISM and QS as non-BBB permeant, whereas ProTox-3.0 classified BBB-barrier toxicity as active for ISM (0.55) and QS (0.64). This difference may reflect variations in the underlying computational models and prediction frameworks. SwissADME’s BOILED-Egg model estimates passive BBB permeation primarily from lipophilicity (WLOGP) and topological polar surface area (TPSA), whereas ProTox-3.0 uses an independently developed ML model based on a distinct training dataset. Thus, “BBB permeant” in Table 5 refers to pharmacokinetic BBB permeability, while “BBB-barrier” in Table 6 represents a separate toxicity endpoint. The Active predictions (0.55 and 0.64) should therefore not be interpreted as direct evidence of BBB permeability or CNS exposure.

### 3.6. Comparative Assessment of ISM and QS

A direct comparison of ISM and QS revealed that ISM more frequently causes DNA interaction-related toxicity and has poor solubility and better predicted bioavailability, whereas QS exhibits greater physicochemical flexibility but still poses significant risks of oral toxicity and mutagenicity. These results highlight how variations in molecular structure lead to different computational toxicity and pharmacokinetic profiles. Importantly, such in silico comparisons provide mechanistic insights at the molecular-property level without implying experimental causation [30]. Previous studies have reported increased apurinic/apyrimidinic DNA sites in mammalian cells following ISM exposure, confirming its genotoxic potential, while nano-formulation substantially decreased DNA damage [7]. Despite long-term clinical use, the exact molecular mechanisms underlying the efficacy, toxicity, and resistance of both ISM and QS remain incompletely understood. Genetic and genomic studies indicate that resistance is multifaceted and related to changes in how drugs are handled inside cells [31], emphasizing the value of in silico studies as tools to generate hypotheses rather than providing definitive mechanistic explanations.

## 4. Conclusions

This study demonstrates that ISM exhibits superior target engagement and conformational stability compared with QS. ISM showed a more favorable binding energy (−8.4 kcal·mol^−1^), formed multiple stabilizing interactions (hydrogen bonds, π–π stacking, hydrophobic contacts) with key active-site residues, and maintained a more stable ISM–protein complex than that of QS. ISM also displayed improved drug-like characteristics, including compliance with Lipinski’s Rule of Five, a lower molecular weight (496.01 g·mol^−1^), a higher predicted bioavailability score (0.55 versus 0.11 for QS), and was not predicted to be a P-glycoprotein substrate. Despite these favorable findings, ADMET analysis flagged high gastrointestinal absorption, limited pharmacokinetic properties, and potential toxicity. Together, the computational and experimental data indicate that ISM has promising trypanocidal target specificity and binding stability but is limited by suboptimal absorption and safety concerns. Therefore, further development should focus on structural optimization to improve pharmacokinetics and absorption, safer formulation, or targeted delivery strategies to realize ISM’s therapeutic potential.

## Figures and Tables

**Figure 1:**
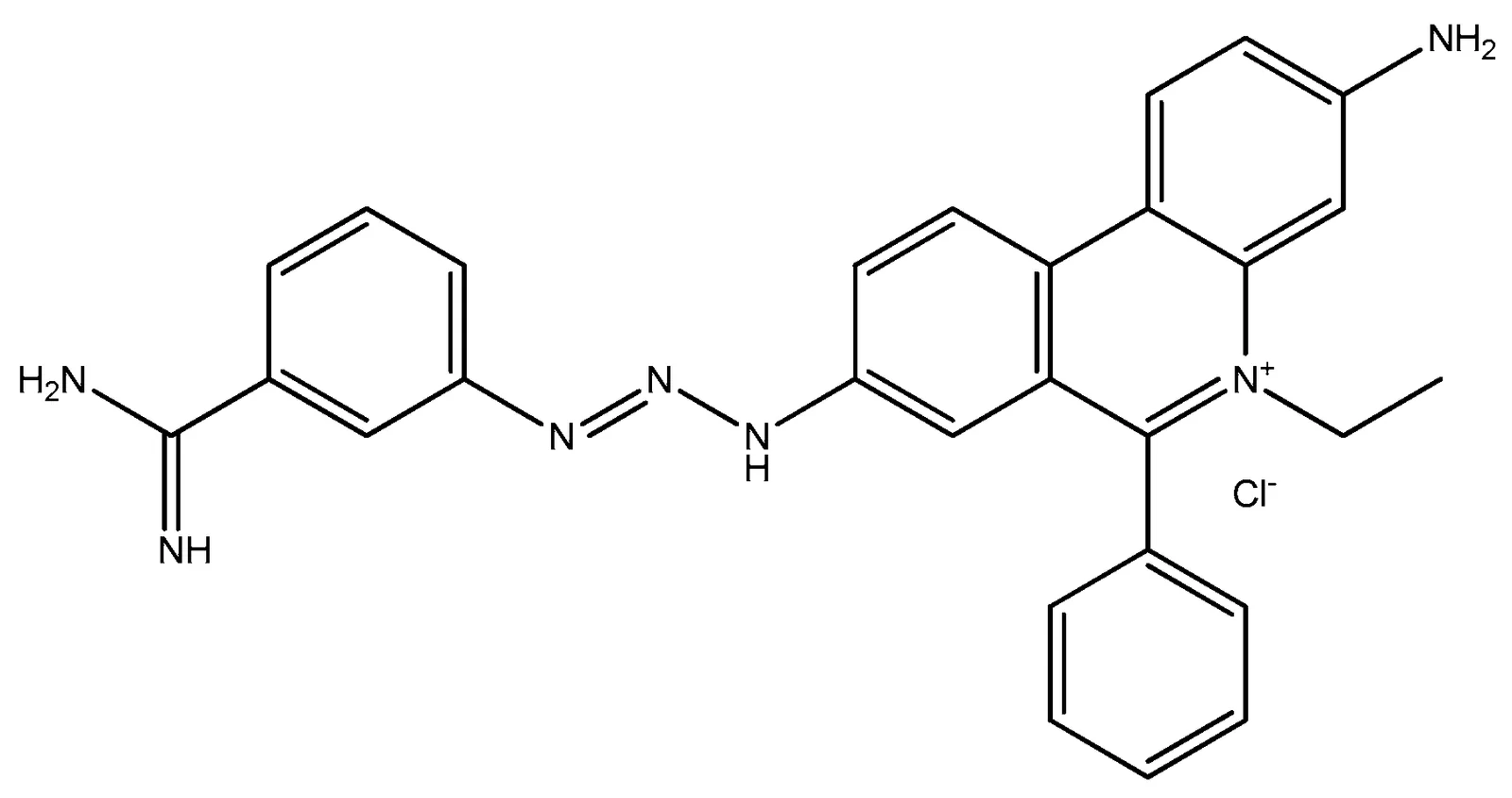
Isometamidium chloride structure.

**Figure 2:**
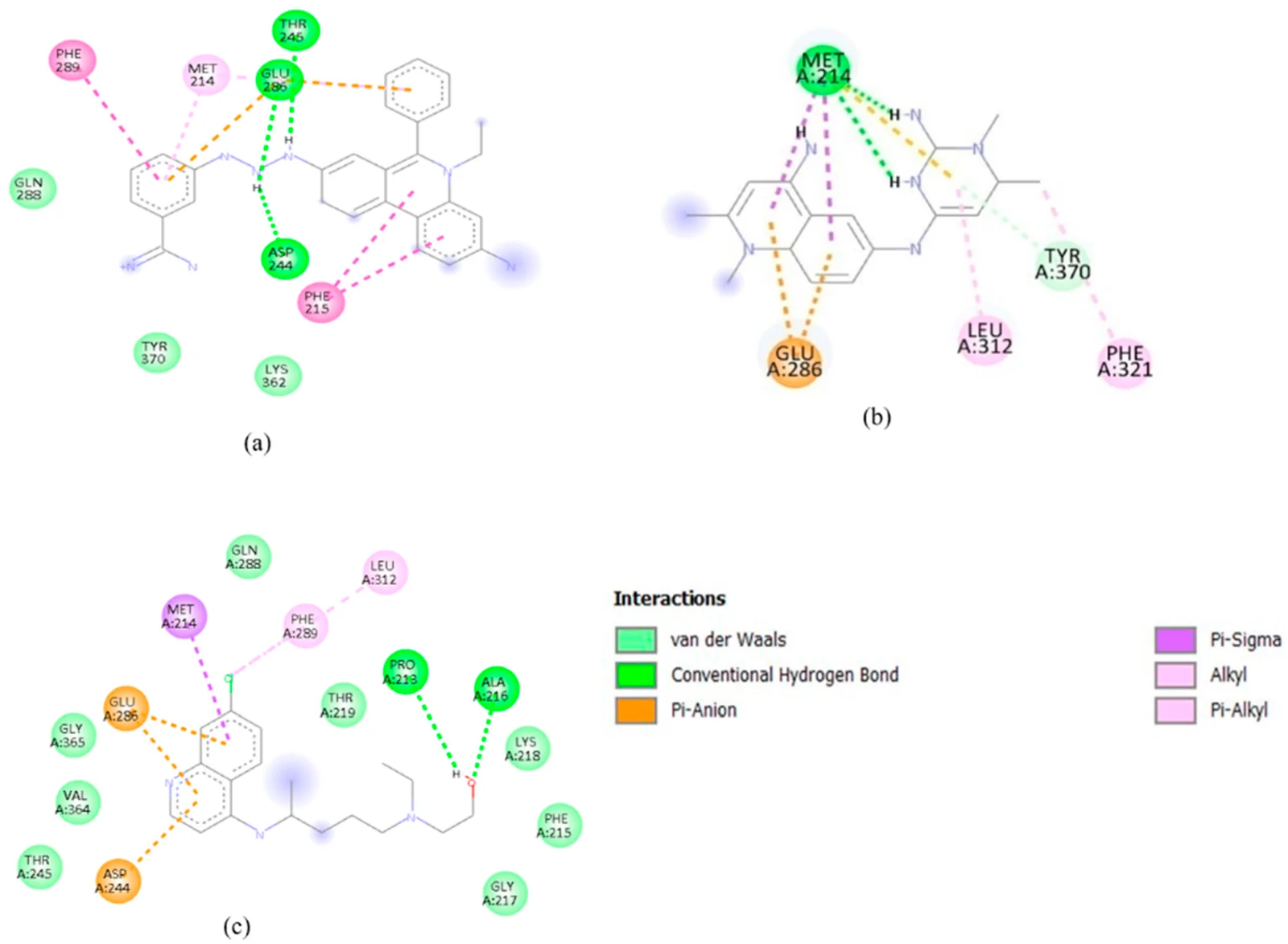
Molecular interaction profiles of (**a**) ISM, (**b**) QS, and (**c**) HCQ with target protein (5FUW), showing key binding interactions. Dashed lines indicate the interactions of the ligand with the key amino acid residues of the receptor.

**Figure 3:**
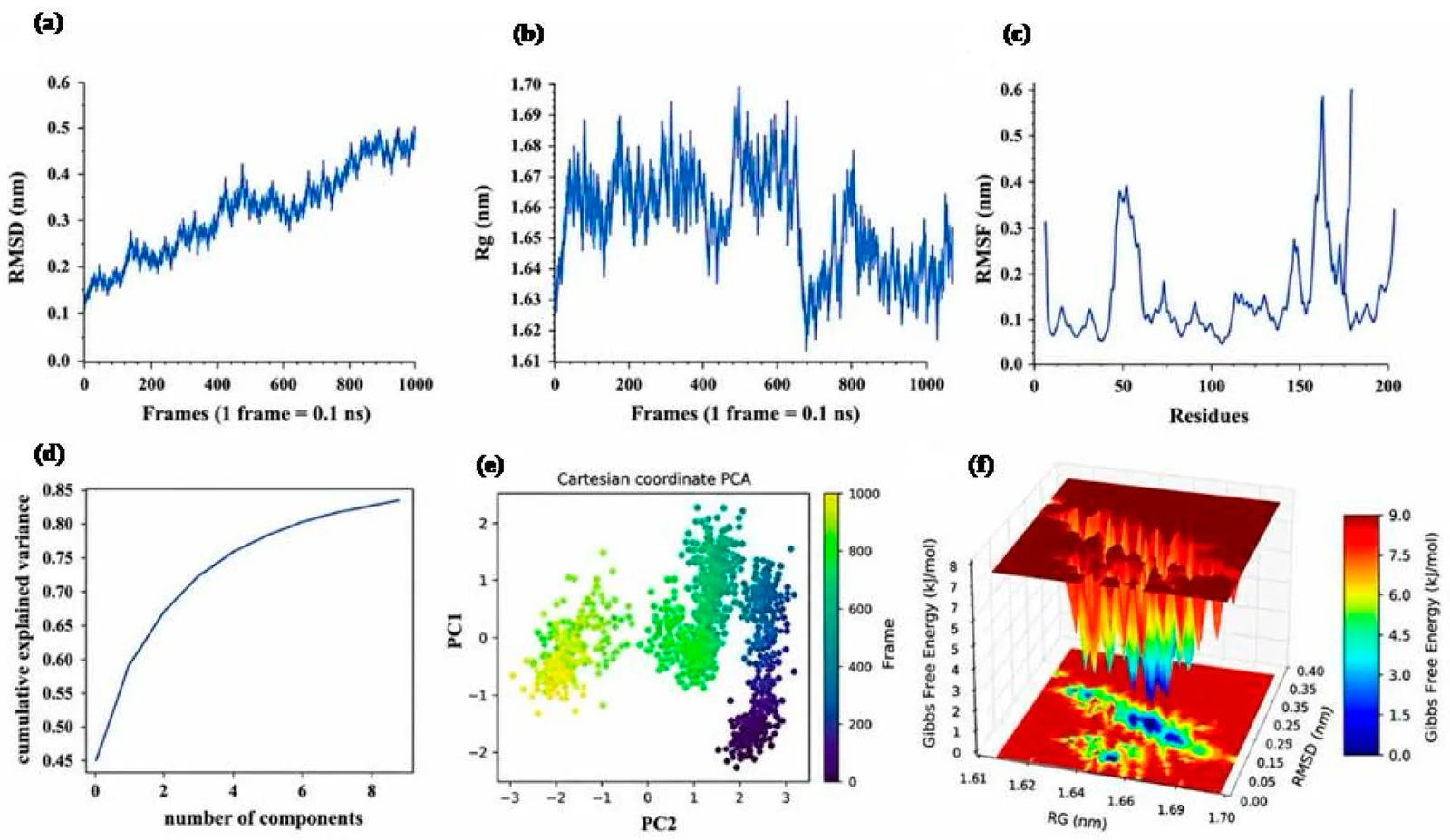
ISM-5FUW results: (**a**) RMSD; (**b**) Rg; (**c**) RMSF; (**d**) cumulative explained variance of principal component analysis (PCA) exponential; (**e**) Cartesian coordinate PCA; (**f**) free energy landscape.

**Figure 4:**
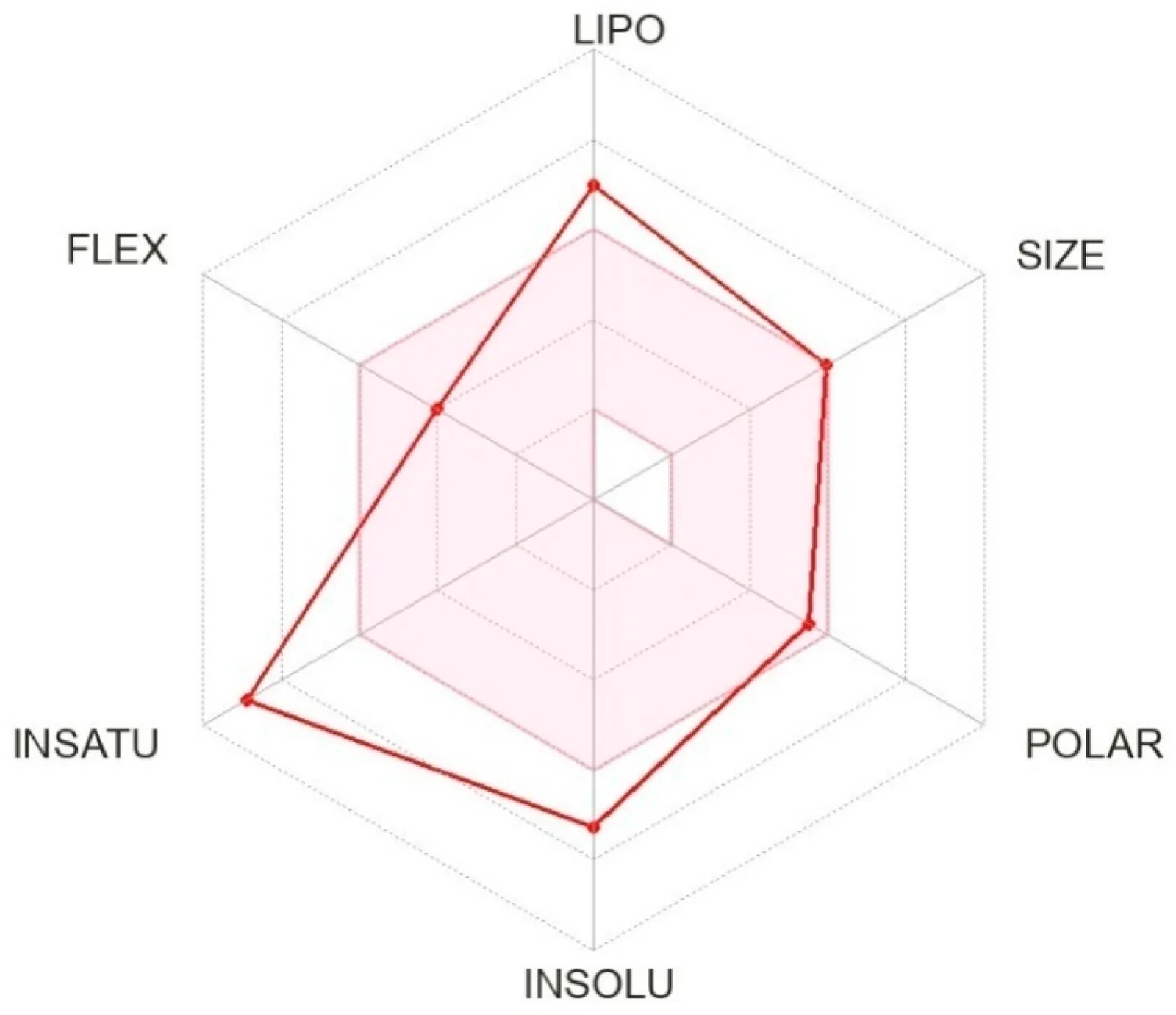
The shaded area indicates the ideal physicochemical range for oral bioavailability, while the red line shows the position of the studied drug. LIPO = lipophilicity; SIZE = molecular weight; POLAR = polarity represented by TPSA; INSATU = insaturation indicated by Fraction Csp3; and FLEX = flexibility, based on the number of rotatable bonds.

**Figure 5:**
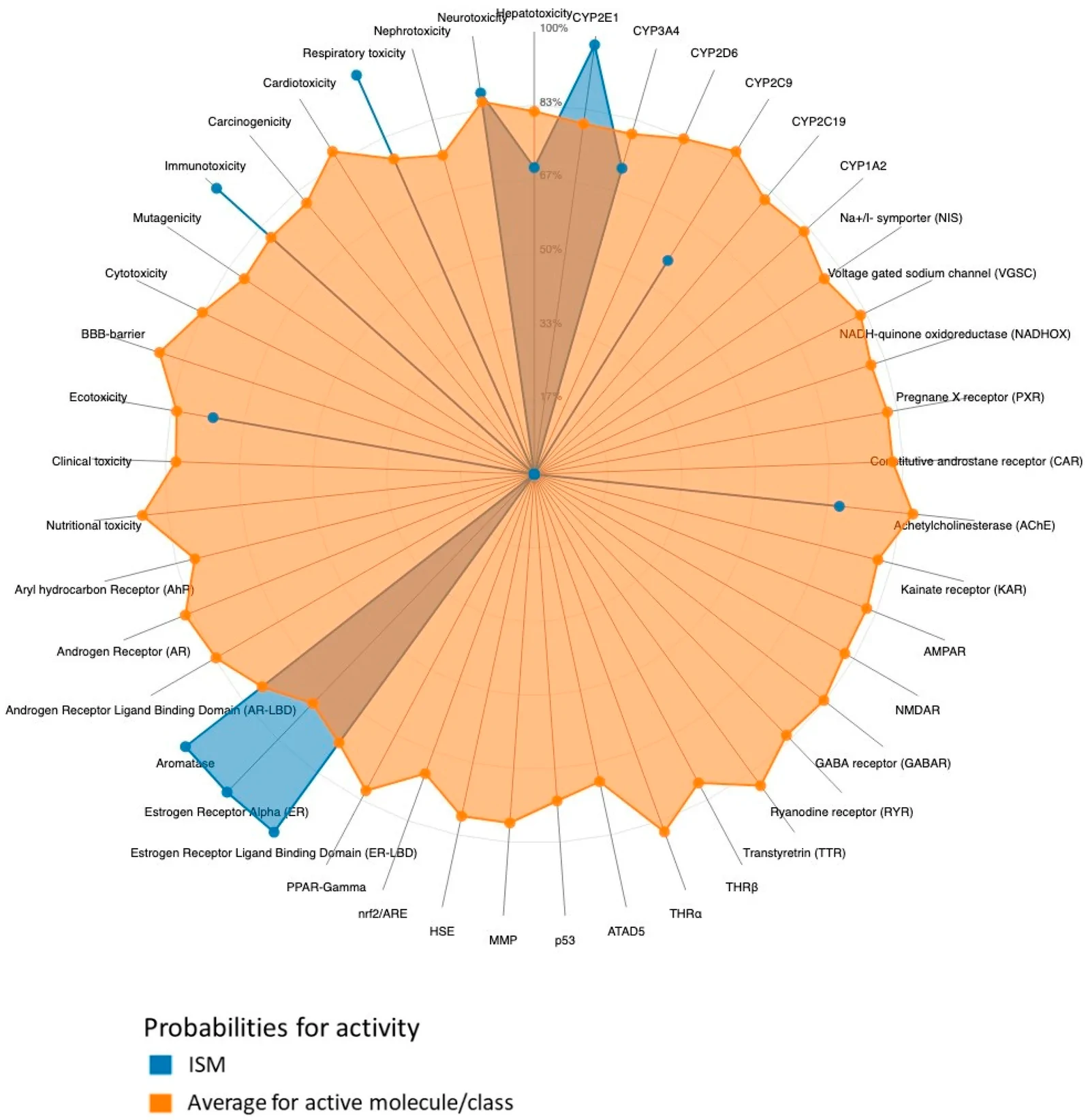
Toxicity radar chart showing the predicted toxicity profile of ISM compared to other molecules.

**Table 1:** Comparative molecular docking analysis of ISM, QS and HCQ.

Drug	Docking Score (kcal/mol)	Key Residues	Binding Strength
ISM (Present Study)	−8.4	Met214, Glu286, Phe289, Thr245, Asp244	Stronger
QS	−5.6	Met214, Glu286, Phe321, Tyr370, Leu312	Moderate
HCQ (Standard)	−8.8	Met214, Glu286, Phe289, Leu312, Asp244	Strongest

**Table 2:** Comparative interaction profile of ISM, QS and HCQ.

Parameter	ISM	QS	HCQ
Binding Affinity	High	Moderate	Highest
π–π Stacking	Strong	Moderate	Limited
Electrostatic Interaction	Strong	Mild	Moderate
Structural Rigidity	High	Moderate	Moderate
Conformational Flexibility	Low	Moderate	High

**Table 3:** Molecular descriptors and physicochemical properties of ISM and QS.

Parameter	ISM	QS
Molecular Formula	C28H26ClN7	C19H28N6O8S2
Molecular Weight (g/mol)	496.01	532.59
Heavy Atoms	36	35
Aromatic Atoms	26	16
Rotatable Bonds	6	3
H-Bond Acceptors	3	10
H-Bond Donors	4	3
Fraction Csp3 (Fsp3)	0.07	0.32
Molar Refractivity	151.21	130.22
TPSA (Å^2^)	116.52	232.89
Consensus Log P	4.17	0.02
LogS (Solubility)	−6.57/−8.13/−9.77	−3.95/−4.69/−2.99
Bioavailability Score	0.55	0.11
Synthetic Accessibility	3.73	3.9

**Table 4:** Drug-likeness assessment of ISM and QS.

Rule Applied	ISM—No. of Violations	ISM—Rule Followed?	QS—No. of Violations	QS—Rule Followed?	Inference
Lipinski	0	Yes	2	No	ISM complies fully; QS fails due to high MW and excess heteroatoms, indicating poorer oral suitability.
Ghose	2	No	2	No	Both compounds violate Ghose criteria: QS due to high MW/MR, ISM due to lipophilicity and refractivity.
Veber	0	Yes	1	No	ISM meets permeability criteria; QS fails due to high TPSA affecting absorption.
Egan	0	Yes	1	No	ISM shows an acceptable bioavailability range; QS exceeds the TPSA limit, reducing permeability.
Muegge	1	No	1	No	Both show one violation: QS due to high TPSA, ISM due to lipophilicity constraint.

**Table 5:** Predicted pharmacokinetic properties of ISM and QS.

S. No.	Parameter	ISM	QS	Inference
1	GI absorption	High	Low	ISM shows better oral bioavailability.
2	BBB permeant	No	No	Neither compound penetrates the CNS.
3	P-gp substrate	No	No	Lower risk of efflux-mediated resistance for both.
4	CYP1A2 inhibitor	Yes	No	ISM has a higher drug–drug interaction risk.
5	CYP2D6 inhibitor	No	No	No CYP2D6 interaction predicted for either.
6	CYP3A4 inhibitor	No	No	No major CYP3A4 metabolic interaction.
7	CYP2C19 inhibitor	Yes	No	ISM shows metabolic interaction potential.
8	CYP2C9 inhibitor	No	No	No CYP2C9 interaction for either.
9	Log Kp (cm/s)	−4.91	−9.88	QS has lower skin permeability than ISM.

**Table 6:** Toxicity comparison of ISM and QS with Tox21 dataset compounds.

Parameter	ISM	QS
LD50 (mg/kg)	100 mg/kg (Class III)	1600 mg/kg (Class IV)
Hepatotoxicity	Inactive (0.59)	Inactive
Neurotoxicity	Active (0.67)	Not Reported
Nephrotoxicity	Inactive (0.61)	Not Reported
Respiratory Toxicity	Active (0.77)	Not Reported
Cardiotoxicity	Inactive (0.87)	Not Reported
Carcinogenicity	Active (0.60)	Inactive (0.51)
Immunotoxicity	Inactive (0.83)	Active (0.98)
Mutagenicity	Active (0.66)	Active (0.64)
Cytotoxicity	Inactive (0.58)	Inactive (0.64)
BBB Penetration	Active (0.55)	Active (0.64)
Ecotoxicity	Inactive (0.53)	Not Reported
Clinical Toxicity	Inactive (0.62)	Not Reported
Nutritional Toxicity	Inactive (0.71)	Not Reported
PAINS Alerts	2	1
Brenk Alerts	6	3

## Data Availability

The original contributions presented in this study are included in the article. Further inquiries can be directed to the corresponding author.
